# Reconsidering the Use of Population Health Surveys for Monitoring of Mental Health

**DOI:** 10.2196/48138

**Published:** 2023-11-23

**Authors:** Jorge Arias de la Torre, Gemma Vilagut, Amy Ronaldson, Ioannis Bakolis, Alex Dregan, Fernando Navarro-Mateu, Katherine Pérez, Anna Szücs, Xavier Bartoll-Roca, Antonio J Molina, Matilde Elices, Víctor Pérez-Solá, Vicente Martín, Antoni Serrano-Blanco, Jose M Valderas, Jordi Alonso

**Affiliations:** 1 Care in Long Term Conditions Research Division King's College London London United Kingdom; 2 Centro de Investigación Biomédica en Red de Epidemiología y Salud Pública (CIBERESP), Instituto de Salud Carlos III Madrid Spain; 3 Department of Biomedical Sciences, Universidad de León León Spain; 4 Health Services Research Group Hospital del Mar Medical Research Institute (IMIM) Barcelona Spain; 5 Institute of Psychiatry, Psychology, and Neuroscience (IoPPN) King's College London London United Kingdom; 6 Agència de Salut Pública de Barcelona (ASPB) Barcelona Spain; 7 Institut d’Investigació Biomèdica Sant Pau (IIB SANT PAU) Barcelona Spain; 8 Department of Medicine National University of Singapore (NUS) Singapore Singapore; 9 Centro de Investigación Biomédica en Red de Salud Mental (CIBERSAM) Madrid Spain; 10 Hospital del Mar Medical Research Institute (IMIM) Barcelona Spain; 11 Institut de Recerca Sant Joan de Déu Parc Sanitari Sant Joan de Déu Barcelona Spain; 12 University of Exeter Exeter United Kingdom; 13 Department of Medical and Life Sciences Universitat Pompeu Fabra (UPF) Barcelona Spain

**Keywords:** mental health, public heath, burden, health surveys, monitoring, status, data collection, electronic health record, challenges, assessment tool, population, population health survey

## Abstract

Monitoring of the mental health status of the population and assessment of its determinants are 2 of the most relevant pillars of public mental health, and data from population health surveys could be instrumental to support them. Although these surveys could be an important and suitable resource for these purposes, due to different limitations and challenges, they are often relegated to the background behind other data sources, such as electronic health records. These limitations and challenges include those related to measurement properties and cross-cultural validity of the tools used for the assessment of mental disorders, their degree of representativeness, and possible difficulties in the linkage with other data sources. Successfully addressing these limitations could significantly increase the potential of health surveys in the monitoring of mental disorders and ultimately maximize the impact of the relevant policies to reduce their burden at the population level. The widespread use of data from population health surveys, ideally linked to electronic health records data, would enhance the quality of the information available for research, public mental health decision-making, and ultimately addressing the growing burden of mental disorders.

## Introduction

In line with the motto of World Mental Health Day on October 10, 2022, “Make Mental Health and Well-Being for All a Global Priority,” mental health has become a public health and research priority in different countries worldwide. In the United States and the United Kingdom, for example, the White House and the UK Health Security Agency have included mental health as one of its corporate priority areas on their health and research strategies [1,2]. Such prioritization processes depend on the availability of data and on the quality of the evidence for a specific context and period. However, not all the data are considered equally relevant for this prioritization, and the evidence derived from population health surveys is often considered to be of lower quality than the evidence from other data sources, such as electronic health records (EHRs).

Population health surveys are observational studies designed for research and public health purposes, aimed to obtain a representation of the distribution of different health conditions and the association of this distribution with sociodemographic, lifestyle, and other factors in well-defined populations. Since the second half of the previous century, these surveys have been carried out in different countries worldwide (eg, the National Health Interview Survey [NHIS] in the United States since 1957 [3] and the Health Survey for England in the United Kingdom since 1991 [4]). Due to their population-based nature (ie, based on representative as opposed to convenience sampling), data from these surveys may constitute a helpful resource to determine the burden of different mental health problems in the community, to identify vulnerable populations or population groups, and to inform the planning of health care resources and the development of health policies and targeted preventive measures [5]. Additionally, it must be highlighted that individual-level data from multiple health surveys worldwide, particularly from middle- and high-income countries, are publicly available for researchers to access and use (eg, data from the NHIS [3] or the National Health and Nutrition Examination Survey in the United States [6] and data from the European Health Interview Survey [EHIS] [7] or the European Social survey in Europe [8]). Some examples of the use of data from population health surveys for these purposes include the use of the data from the World Mental Health Surveys as a primary source to calculate the prevalence of major depression internationally [9], the identification of differences in depression among religious groups in Europe using data from the European Social Survey [10], and monitoring of the mental health and suicide prevention reforms in Australia [11]. While the use of data from population health surveys has been successfully implemented for these purposes, these data need to meet certain requirements, including the use of valid and reliable measures for the assessment of mental disorders and the selection of representative samples.

In this context, we propose that extending the use of data from population health surveys would have a considerable impact on improving the evidence base of public mental health decision-making and, hence, could be instrumental in reducing the growing burden of mental disorders in the community.

## Public Mental Health and the Use of Population Health Surveys

Monitoring of the mental health status of the population and assessment of its determinants (eg, sociodemographic factors, lifestyle habits, and use of health services) are 2 of the pillars of public mental health [12-14], and the use of harmonized data from multiple sources could be instrumental to support them.

If the quality of the data could be guaranteed, the monitoring of mental health at the population level should be based on all the available data. For example, the Global Burden of Disease study integrates data from several sources, including data from more than 65,000 household health surveys and 87,000 medical records [15]. Although this can be considered the ideal scenario and there is growing interest on the data from population health surveys, particularly due to their potential to reach different population groups with low accessibility by using digital surveys, these data have been used less frequently than data from EHRs for monitoring and informing public mental health decision-making [16,17]. This is the case in several countries, such as Italy and the Netherlands, where EHRs have been used, for example, to monitor the impact of the COVID-19 pandemic on the mental health of the general population [18] and to monitor mental health conditions in children [19], respectively.

One controversial aspect related to the use of data from population health surveys for the monitoring of mental health is the type of mental health measures included within them. Population health surveys commonly include self-reported measures (eg, the 8-item version of the Patient Health Questionnaire) that are considered less valid and reliable than clinical interviews. However, it must be highlighted that, in contrast with other medical conditions, and because of the subjective and self-reported nature of most of the symptoms of mental disorders [20,21], self-reported tools (eg, the 8-item version of the Patient Health Questionnaire) represent valid and reliable measures at the population level (group or aggregate level) and, hence, can be used for timely monitoring and identification of (vulnerable) groups of interest.

Timely assessment and identification of factors related to mental disorders is another strength of the data captured by population health surveys. These surveys usually include within their questionnaires very rich information about sociodemographic and environmental determinants (eg, education and air pollution) and lifestyle habits (eg, diet or physical activity), constituting a valuable resource to assess their potential relationship with mental health. One example of the use of data from population health surveys to assess sociodemographic determinants of mental health is the use of data from the second and third waves of the EHIS (EHIS-2 and EHIS-3, respectively) to identify differences by country in the prevalence of depression across 27 European countries (with a higher prevalence: 1.8 times higher in Germany and 1.5 times higher in Luxembourg relative to the rest of Europe) [22,23]. Additionally, some population health surveys capture data about the use of mental health services (eg, the number of primary care consultations during a specific time), which is a potentially useful resource to inform the planning of public mental health resources [13,24,25]. This information has been previously used to investigate the unmet need of mental health care [9,24] and is particularly relevant for assessing vulnerable population groups that usually have higher rates of mental disorders, lower access to these services or, due to their circumstances, do not use these services when needed (eg, people experiencing homelessness) [26].

## Synergistic Relevance of the Data From Population Health Surveys and EHRs

While the use of population health surveys is suitable for different public mental health purposes, in some cases, data from EHRs could be a better option due to their potentially higher completeness and representativeness, the inclusion of richer relevant clinical data, and the possibility of following up with their participants over time (Table 1).

**Table 1:** General characteristics of population health surveys and electronic health records (EHRs).

	Population health surveys	EHRs
Population included	General population	Clinical population
Representativeness	Household samples to quota samples	Usually public providers
Type of design	Usually cross-sectional	Longitudinal
Assessment of mental disorders	Usually self-reported	Usually diagnostic codes
Range of mental disorders usually considered	Limited	Wide
Other relevant variables	Sociodemographic factors, lifestyle habits, and use of health services	Only clinical variables
Cost	Low	High

One advantage that EHRs have over population health surveys is their potentially higher external validity. In contrast with surveys, which are usually carried out in samples with different degrees of representativeness, data from EHRs are more likely to include entire populations, such as the Hospital Episodes Statistics in England, in which more than 99% of attendees of mental health services from the National Health Service of the United Kingdom are captured [27]. Besides, data from EHRs usually capture high-quality clinical information, such as information about diagnostics (eg, International Classification of Diseases [ICD] codes), treatments (eg, prescribed medication), and other clinical aspects of care (eg, the number of consultations in a specific service), positioning them as the most suitable alternative for the assessment of the use of mental health services and to inform their planning [16,17.24]. Paradoxically, it should be noted that despite the quality of the data from EHRs (particularly to capture severe mental disorders), medical records are not designed for research purposes and focus on clinical populations (ie, not in the general population), thus limiting their generalizability to the general population and even more so to vulnerable population groups less likely to access these services [26]. Additionally, data from EHRs are limited by data capturing systems. However, given the potential differences in codification systems (eg, mental disorders codified using different ICD versions [21]) and across studies, achieving semantic interoperability is a key aspect to consider when leveraging data across multiple data sets [28]. As a consequence, certain relevant outcomes (eg, drug abuse) may be poorly documented and need to be validated for research, or they reach only public mental health service users, thus limiting their generalizability to the growing population groups using private health care services.

Another advantage of EHRs over population health surveys is the possibility of long-term follow-up of participants. While population health surveys are often cross-sectional and anonymous (or anonymized) and participants cannot be followed up over time, the inclusion within EHRs of variables that facilitate the identification of participants and date variables (eg, dates of admission or follow-up consultations) make it possible to follow up with them over time. This is particularly important for correct estimation of the incidence of different health problems, the rates of recovery or relapse of some disorders over time, or the impact on mental health over time on different factors and the establishment inferences about potential causal associations [29]. However, there is an increasing interest in longitudinal surveys, in panel data with repeated assessments of the same individuals over time (eg, the British Cohort Study in the United Kingdom) [30,31], and in the inclusion of variables within the survey questionnaires that allow the follow-up of their participants over their life course (eg, participant identification codes). Thus, while EHRs could be considered currently more suitable for the timely assessment of clinical aspects of mental disorders, complementing their data with those from longitudinal surveys could enhance and enrich such assessment [32,33].

Despite the abovementioned differences between the data from population health surveys and those from EHRs for monitoring of mental health, the complementary nature of these data sources must be highlighted. One successful example of their complementarity for the study of mental disorders has been reported in Denmark [32], where a shared identification number assigned to the individuals included in their information systems allows the linkage between population health surveys and EHRs [33]. The extensive linkage of individual data in the Danish population (including population health surveys; national records from hospitals, clinics, pharmacies, and death registries; and various other public and private data sources) synergistically enhanced the data’s overall quality for the assessment and monitoring of mental health conditions.

## Moving Forward Toward the Generalization of the Use of Population Health Surveys in Public Mental Health

Given all the potential benefits and applications indicated herein, widespread use of population health survey data (ideally linked with data from high-quality EHRs) could be helpful both for research purposes and to enhance decision-making in public mental health. Their widespread use might be achieved through not only the deployment of large, address-based probability-sampled surveys, similar to the deployment of the NHIS in the United States [3], but also encouraging individual research groups to join forces and carry out large-scale population health surveys or leverage the ones already implemented. However, in order to guarantee adequate and appropriate use of data from population health surveys, the development of a common usage framework and enhance their interoperability with other data sources remains essential.

The development of a common usage framework of data from population health surveys must be in line with widely recognized initiatives such as the GRADE (Grading of Recommendations Assessment, Development and Evaluation) Evidence to Decision (EtD) framework [34], and will need to include unified protocols and guidelines for data collection, data analysis, and interpretation. For data collection, guidelines must consider both the tools used for monitoring of mental health and the minimum set of general domains (eg, socioeconomic and health service use) and specific factors (eg, educational level attained and number of primary care consultations during the last year) included within any population health survey.

It should be also mentioned that the data collection process and the potential biases related to the representativeness of the data and their quality are also key challenges. The use of multistage sample designs and data verification techniques, for instance, would enhance the validity of data from population health surveys for both research and public mental health purposes. Additionally, for the correct interpretation of their results, a set of valid and reliable standard measures for the assessment of a broad range of mental disorders needs to be established, and their cross-cultural equivalence in different contexts should be ensured [10]. Therefore, the development of a common usage framework for the use of population health surveys for monitoring mental health would improve the suitability of their data for this purpose, enhance the comparability of their results and, consequently, increase their impact on public mental health decision-making.

Finally, given the complementing advantages of the data from population health surveys and those from EHRs, another key step forward is to promote the interoperability of these 2 sources. However, to achieve interoperability, it will be necessary to systematically include variables within their data sets, which facilitate the identification and linkage of individuals across them while also ensuring anonymity. This linkage will be helpful to bring together their strengths and potentially improve the accuracy and relevance of both EHRs and health surveys as tools for monitoring mental health at the population level [35]. However, due to the substantial variation between countries in data protection laws, systematically including these types of variables could be challenging, particularly in the case of international surveys, such as the World Mental Health Surveys or EHIS. Hence, promoting the implementation of shared international regulations about data linkage and anonymization could be a helpful way to enhance the quality of administrative health data for monitoring the health status of the population.

## Opportunities and Implications of the Use of Population Health Surveys

Monitoring of mental disorders at the population level, identifying their determinants, and determining their association with health care usage are key components for effective mental health prevention. Balancing their advantages and limitations, data from population health surveys are instrumental in addressing these challenges. However, to guarantee the adequate use of these data, there are different aspects that should be considered, including their specific limitations and challenges, the potential adoption of a common and shared framework for their use, and their interoperability with other data sources, such as EHRs, using shared identification variables. Promoting the use of linked data from population health surveys, EHRs, and other public and private data sources could enhance the quality of the information available for public mental health decision-making, particularly in middle- and high-income countries and ultimately improve the planning of mental health resources and maximize the impact of relevant policies to reduce the burden of mental disorders.

## Abbreviations

EHIS: European Health Interview Survey
EHIS-2: second wave of the European Health Interview Survey
EHIS-3: third wave of the European Health Interview Survey
EHR: electronic health record
EtD: Evidence to Decision
GRADE: Grading of Recommendations Assessment, Development and Evaluation
ICD: International Classification of Diseases
NHIS: National Health Interview Survey
